# Teacher Agency in Ghanaian Schools: Impact of Career Choice Motivations and Perceptions of the Teaching Profession

**DOI:** 10.3390/bs15070895

**Published:** 2025-06-30

**Authors:** Francis Adams, Qiong Li, Hu Mu

**Affiliations:** 1 School of Foreign Languages and Cultures, Nanjing Normal University, Nanjing 210046, China; francisadams69@yahoo.com (F.A.); humu@njnu.edu.cn (H.M.); 2 Faculty of Education and Center for Teacher Education Research, Key Research Institute of the Ministry of Education, Beijing Normal University, Beijing 100875, China

**Keywords:** teacher agency, motivation, perceptions, basic school teachers, Ghana

## Abstract

This study explored teacher agency and its impact on the motivation and perceptions of the teaching profession. This study employed a cross-sectional survey design, in which questionnaires were administered to a sample of 574 basic school teachers in Ghana. Structural equation modeling (SEM), a multivariate statistical technique for analyzing complex relationships among latent constructs, was employed to examine the direct, indirect, and moderated effects among the study variables, thereby ensuring accurate and reliable results. This study found that the motivation and perception factors have a strong positive impact on teacher agency. The moderator analysis showed that intrinsic career value was a significant predictor of teacher agency for both male and female teachers. The results also indicated the significant moderating effect of age on the relationship between intrinsic career value and teacher agency. Additionally, the findings revealed that perception factors partially mediate the relationship between motivation factors and teacher agency. However, this study is limited by its cross-sectional design and focus on public basic school teachers in Ghana, suggesting the need for future research to include longitudinal approaches, broader geographic representation, and private school contexts. Finally, the theoretical and practical implications are addressed.

## 1. Introduction

Teacher agency is a crucial aspect of the teaching profession that is progressively acknowledged within the educational field (Priestley et al., 2015). An agentic teacher is determined to enhance the learners’ advancement and their capacity to handle the growing intricacies of the teaching career efficiently (Long et al., 2017). The agentic teacher is transformative, ensuring purpose and consistency, and motivates all pupils while protecting against the influence from the media, education departments, and other interested individuals. In teacher agency studies, a teacher plays a vital role in the school environment (Toom et al., 2015). As such, teachers must view themselves as responsible pedagogical specialists capable of providing help and resources to students, parents, and peers while facing challenging situations. Even though teachers use agency for various reasons, including pedagogy, morality, and transformation (Yang, 2015; Molla & Nolan, 2020), the current study focuses on teacher agency from the perspective of the teaching process.

Despite extensive exploration of the concept of agency across various disciplines, empirical educational research and theoretical advancement focused explicitly on teacher agency are scarce, particularly in the literature on educational change (Priestley et al., 2012; Tao & Gao, 2017; Leal & Crookes, 2018). Nevertheless, there are still prospects for additional contributions to the existing studies. Three primary research gaps are particularly pertinent to this study. First, most studies have investigated the relationship between teacher agency and other factors (e.g., student engagement, student achievement, job satisfaction, teacher identity, and professional development), and have applied psychosocial theory for a deeper, broader view of teacher agency in developed countries, such as the United Kingdom (Clarke & Kennedy, 2024), United States (Hawthorne-Kocak, 2021), European countries (Sannino, 2010; Kusters et al., 2023; Juutilainen et al., 2024), and South Korea (Namgung et al., 2020), as well as developing countries, such as Türkiye (Kondakci et al., 2017; Ersöz, 2021), Vietnam (Vu, 2020), Saudi Arabia (Ahmad & Shah, 2022), and China (Liu et al., 2016). There are limited studies on the topic because most were conducted in developed countries, and only a few studies have been conducted in Sub-Saharan Africa.

Second, the concept of teacher agency is centered on the idea that it significantly enhances teacher effectiveness (Vähäsantanen, 2015). Nonetheless, there is insufficient empirical evidence that teacher career motivation and perceptions of the teaching profession are associated with teacher agency or that gender and age act as moderators between the variables. Again, there is a lack of evidence regarding the mediating role of perception factors between motivation and teacher agency.

Third, the analysis and synthesis of the literature show that earlier studies have predominantly focused on qualitative research and the variables that impact teacher agency and techniques for promoting teacher agency (e.g., Kusters et al., 2023; Juutilainen et al., 2024; Rushton & Bird, 2024). However, there is a lack of major quantitative research studies that establish teacher agency and its impact on the motivation and perceptions of the teaching profession. To fill these research gaps, this study explores teacher agency and its impact on the motivation and perceptions of the teaching profession in the developing country of Ghana.

Applying self-determination theory (SDT), this study constructed a conceptual framework to examine teacher agency and its impact on the motivation and perceptions of the teaching profession in Ghana. This study employed survey data from 574 basic school teachers (kindergarten, primary, and junior high school) in Ghana to analyze the research hypotheses using structural equation modeling (SEM). The following three questions are addressed:

(a) How do motivation and perception factors influence teacher agency among Ghanaian basic school teachers?
(b) How does perception mediate the relationship between motivation factors and teacher agency?
(c) How does gender and age moderate the influence of the proposed predictors on teacher agency among Ghanaian basic school teachers?

This study makes some contributions. First, using the SEM approach, this study adds to the knowledge of the existing policies and the limited literature that addresses teacher agency and its impact on the motivation and perceptions of the teaching profession in Ghana, a developing country. Second, by extending SDT, this study determines the relationship between teacher agency, motivation factors, and perceptions. Third, this study aimed to determine whether gender and age moderate the influence of the proposed predictors on teacher agency. Fourth, this study examined whether perception factors mediate the relationship between the motivation factors and teacher agency. Finally, this study provides the government and policymakers with strategies to enhance the appeal of the teaching profession to highly skilled individuals and to boost current teachers’ motivation. The findings of this research enlighten the stakeholders within the education sector on the substantial influence of teacher agency and direct actions to recognize and enhance its potential.

## 2. Literature Review

### 2.1. Ghana’s Education System

Ghana’s education system is deeply influenced by its British colonial heritage, which established its foundational structure and curriculum that persisted post-independence in 1957. The pre-tertiary education framework comprises basic education, i.e., two-year kindergarten, followed by a six-year primary school, and a three-year junior high, culminating in the Basic Education Certificate Examination (BECE). Following this, the second cycle includes a three-year program at either a senior high school or an institution offering technical/vocational education and training, where students undertake core and elective subjects aligned with their chosen specialization, ending with the West African Senior School Certificate Examination (WASSCE). Tertiary education encompasses universities, technical universities, and colleges of education, offering various undergraduate and postgraduate programs, with English as the official language of instruction across all levels. Ghana’s tropical climate, with distinct rainy and dry seasons, varies by region. Dry harmattan winds occur from November to March.

Teacher preparation at the basic education level primarily occurs in Colleges of Education (CoE), which increased from 38 in 2014 to 48 due to the government’s acquisition of private institutions to improve infrastructure. These CoE transitioned from offering a three-year diploma program in basic teacher education (Buabeng et al., 2020) to Bachelor of Education (B.Ed.) degrees across multiple specializations. Additionally, the University of Cape Coast and the University of Education, Winneba, serve as key institutions for teacher education, equipping educators with comprehensive subject knowledge and pedagogical skills (MOE, 2018). However, a significant number of graduates from other universities, lacking formal teacher training, are employed as non-professional teachers at various school levels (Anamuah-Mensah & Benneh, n.d.).

According to the MOE (2012), Ghana’s pre-tertiary educators are categorized as professional or non-professional. Professional basic school teachers must possess a diploma-level qualification in basic education from an accredited CoE aimed at ensuring adequate pre-service training and instructional competence (T-TEL, 2018). At the second cycle level, professional status requires an undergraduate degree in education or a relevant discipline, supplemented by a Postgraduate Diploma in Education (PGDE) or the equivalent. Non-professional teachers either hold Senior High School certificates with credits in English and Mathematics or diplomas from polytechnics (now technical universities) or university degrees without teaching certification (MOE, 2012).

### 2.2. Teacher Agency

Teacher agency is the ability of teachers to make choices, act purposefully (Anderson, 2010), and drive positive change in their teaching practice (Hadar & Benish-Weisman, 2019). Pyhältö et al. (2012) explain that the teachers’ professional agency is also represented through their self-efficacy, motivation, and participative abilities.

A study by Van der Heijden et al. (2015) found that agent teachers were highly competent and effective teachers who had a positive impact on students’ learning and well-being. Such teachers who possess a high sense of agency are more likely to actively construct and implement new pedagogical practices (Pyhältö et al., 2014). Molla and Nolan (2020) noted that agency teachers have higher expectations for their students, and employ multiple strategies to address their varied learning needs. The teachers’ participation in decision-making makes a contribution to school leadership through the incorporation of their valuable opinions and suggestions (Brezicha et al., 2020).

The existing research has indicated that agency in teachers might be determined by their skills, knowledge, attitude, and values that have developed based on personal experiences as well as work experience (Pyhältö et al., 2014; Molla & Nolan, 2020; Brezicha et al., 2020). How they interact every day with fellow workers and with the school system is likely to reinforce or interfere with agency (Pyhältö et al., 2012). The teachers who report feeling high social support with students and showing empathy with students also cultivate agency (Van der Heijden et al., 2015), along with promoting shared school values and school administration (Oolbekkink-Marchand et al., 2017).

In developed countries, such as the United Kingdom, the United States, Europe, and South Korea, the dynamics between teacher agency and other factors have changed relatively rapidly (Clarke & Kennedy, 2024; Hawthorne-Kocak, 2021; Sannino, 2010; Kusters et al., 2023; Juutilainen et al., 2024; Namgung et al., 2020). Several reasons have contributed to this trend, such as education reforms (Biesta et al., 2015), the need for professional development (Vähäsantanen, 2015), a move towards student-centered learning (Toom et al., 2015), policy reforms (Sahlberg, 2011), the development of technology (Ertmer & Ottenbreit-Leftwich, 2010), and socio-cultural developments (Goodall & Montgomery, 2014). In developing countries, such as China and India, a rise in education investments has made significant socioeconomic progress. Academic institutions in developing countries also place increasing emphasis on education research, such as teacher agency research (e.g., Lee, 2014; Tao & Gao, 2017; Moitra, 2024).

However, not much has been studied on teacher agency, motivation, and perceptions toward teaching in developing countries in Africa. Given the urgency of Africa’s need for education reforms, a study of teacher agency might provide helpful insight into how to enhance the purpose and motivation among African teachers. This study, conducted in Ghana, bridges this gap by exploring how the motivation and perceptions of the teaching profession are associated with teacher agency.

### 2.3. Theoretical Review

SDT is a wide-ranging theory of human motivation, with a specialization in the inherent tendencies of individuals for self-development and their basic psychological needs (Ryan & Deci, 2017). Kaplan (2014) argues that SDT operates under the belief that the mental state of human beings relies on the essential need for ‘autonomy’, ‘competence’, and ‘relatedness’. According to the theory, individuals who feel their needs are satisfied are likely to exhibit self-regulated and responsive behaviors that are geared toward personal development.

SDT has also been used in a range of research on teacher agency (e.g., Roth et al., 2007; Taylor et al., 2008; Pietarinen et al., 2013; Skaalvik & Skaalvik, 2014; Collie et al., 2012). Specifically, Roth et al. (2007) found that teachers who were supported in autonomy reported greater job satisfaction and well-being. The support type was associated with greater intrinsic motivation and greater professional agency. Skaalvik and Skaalvik (2014) reported a significant connection between the teachers’ self-report of ability, job satisfaction, and motivation. Professional development programs that cater to the teachers’ needs and objectives render them empowered and more confident. Pietarinen et al. (2013) quoted that, when there was a supportive environment by the school leaders who provided teachers with an opportunity to participate in decision-making and facilitated professional development, the effect on the teachers’ perception of agency and job satisfaction was significant. Thus, we employed the SDT as a theoretical lens to investigate the influence of teacher agency on the motivation and perceptions towards teaching in Ghana.

## 3. Hypothesis Development and Conceptual Development

### 3.1. The Relationship Between Motivation Factors and Teacher Agency

Intrinsic career value is the joy, satisfaction, and sense of meaning that individuals derive from their career (Jackson & Tomlinson, 2019).

Teachers who find a sense of meaning in their profession are generally more dedicated, enthusiastic, and engaged in their teaching practices, which leads to improved teaching quality (Fathi et al., 2023). Intrinsic value can enhance the teachers’ potential for building good rapport with students (Oberhauser & Hertel, 2023), establishing a positive and nurturing classroom environment focused on student achievement and well-being. Therefore, this study proposes the following hypothesis:

**H1.**  *Intrinsic career value is positively related to teacher agency.*

A fallback career is a second profession that individuals pursue when their first profession fails. The effectiveness of individuals with fallback professions to teach depends on whether they are motivated, committed, and open to learning how to teach. Their various views and applicable skills can improve their ability to teach (Ertürk, 2023; Agaj et al., 2023). For instance, someone with project management experience may bring organizational and leadership skills to the classroom, improving lesson planning, classroom management, and student engagement (Shah & Bhattarai, 2023). Therefore, this study proposes the following hypothesis:

**H2.**  *Fallback careers are positively related to teacher agency.*

Job security is the stability and guarantee of ongoing employment in a specific profession. Kumar (2023) emphasized that job security positively impacts the teachers’ performance by providing them with stability and tranquility, allowing them to concentrate on their primary duty of imparting knowledge to students. It also promotes loyalty and commitment to the profession (Agbonna et al., 2023). Teachers with secure career paths are more likely to invest in professional development, seek personal growth, and continuously improve their teaching skills (Sario & Villocino, 2023). Thus, we propose the following hypothesis:

**H3.**  *Job security is positively related to teacher agency.*

Social influences pertain to the effects of the surrounding environment, cultural factors, and social interactions on an individual’s cognition, emotions, and actions. Within the domain of education, social influences can exert a substantial impact on teacher efficiency. Social influences can mold the general atmosphere of a classroom, which includes relations between the teachers and the pupils, as well as among the students (Reichert et al., 2018). A positive classroom environment, built on trust, respect, and cooperation, can improve teaching efficiency by promoting student participation, motivation, and learning (Meyer & Turner, 2006). On the other hand, negative social elements, such as disruptive behavior or a lack of classroom cohesion, can impede effective instruction (Babad, 2009). Therefore, this study proposes the following hypothesis:

**H4.**  *Social influences are positively related to teacher agency.*

### 3.2. The Relationship Between Perception Factors and Teacher Agency

An individual’s level of proficiency in a specific subject or discipline determines their capability to teach and impart that knowledge to others (Zaragoza et al., 2023). Teachers who are highly proficient in a discipline possess extensive knowledge and comprehension of the concepts, theories, and applications associated with the subject (Loughran, 2012). From this proficiency, they can explain things clearly and in detail, answer questions adequately, and address challenging topics with confidence. the teachers’ experience teaches them about the newest discoveries and advancements in their chosen field of practice (Stronge, 2018). Therefore, this study proposes the following hypothesis:

**H5.**  *Expertise is positively related to teacher agency.*

Social status refers to an individual’s position within a social hierarchy based on educational background, occupation, income, and public respect (Richards et al., 2023). People with higher social status, for example, professors or specialists, have better opportunities and resources for professional advancement, with the benefits of higher research and contacts that enhance their quality of teaching. More socially valued teachers will be more inclined to build credibility and respect in their classrooms, and make positive contributions to the ways they interact with students and other teachers (Hagenauer et al., 2023). Therefore, this study proposes the following hypothesis:

**H6.**  *Social status is positively related to teacher agency.*

According to Agboola and Offong (2018), salary is critical in attracting and retaining skilled and engaged teachers. Sufficient remuneration provides financial security and job satisfaction, encouraging highly qualified people to enter and remain in teaching (Podolsky et al., 2016). The teachers who are sufficiently compensated financially can focus on their work and invest in professional growth, which makes the classroom run more smoothly. Ololube (2006) further stated that increased earnings improve work motivation and teaching performance since well-paid teachers are more motivated, engaged, and committed to the students’ achievement. Therefore, this study proposes the following hypothesis:

**H7.**  *Salary is positively related to teacher agency.*

Social dissuasion means that people or groups utilize their influence or opposition to discourage specific activities or ideas. Eren and Çetin (2019) stated that social dissuasion can compel teachers to critically look at their teaching practices, beliefs, and approaches. Constructive feedback and counterarguments might prompt the teachers to improve their classroom practices, derive lessons from a diversity of viewpoints, and realistically adapt their approaches to the necessities and interests of their learners (Bienkowski et al., 2012). Through open conversation and adjusting approaches, teachers have the potential to improve their teaching effectiveness in interactions with learners as well as to raise learning outcomes. Therefore, this study proposes the following hypothesis:

**H8.**  *Social dissuasion is positively related to teacher agency.*

Day (2004) reported that the job satisfaction of teachers regarding their profession is usually tied to a strong commitment and love for teaching. Nida and Ningsih (2023) emphasized that teachers who love what they do are more likely to perform it with passion and drive, leading to an aspiration for constant improvement and quality instruction. Satisfaction with the profession also raises job satisfaction, since satisfied teachers tend to have favorable attitudes, greater commitment, and greater job stability (Akhtar et al., 2010). The satisfaction generates a favorable work culture and improved student–teacher relationships, both of which are critical to effective teaching and learning. Therefore, this study proposes the following hypothesis:

**H9.**  *Satisfaction with choice is positively related to teacher agency.*

### 3.3. The Mediating Role of Perception Factors

Perception factors serve as mediators of teacher agency and motivation, influencing the way teachers perceive and respond to motivational forces (Guilloteaux, 2007). Teachers are likely to translate their motivation into action when they believe in the significance of education and see themselves as change agents in the classroom. Self-doubt or dissatisfaction with the educational system, however, would block this transformation. Perceptions also shape the teachers’ responses to external incentives (Inman & Marlow, 2004). For instance, the teachers who see salaries and promotions as symbols of appreciation may be less motivated than those who perceive them as reflections of their worth and impact. Therefore, this study proposes the following hypothesis:

**H10.**  *Perception factors mediate the relationship between motivation factors and teacher agency.*

### 3.4. The Moderating Influence of Gender

Research suggests possible differences in how students perceive and respond to male and female teachers, which can impact their evaluations of teaching effectiveness (Kogan et al., 2010). Research suggests that male individuals in educational and professional settings prioritize being perceived as successful, skilled, and possessing a high social standing. This preference may explain their hesitation in pursuing jobs with a lower social status, particularly those traditionally linked to women’s labor (Thornton et al., 2002). Statistically significant gender disparities were observed in social dissuasion, with men being more prone to reporting discouragement from others while considering a career in teaching (Jugovic et al., 2022). Therefore, this study proposes the following hypothesis:

**H11.**  *Gender moderates the influence of proposed predictors on teacher agency such that the strength of the relationship is different for males and females.*

### 3.5. The Moderating Influence of Age

The influence of various predictors on teacher agency varies across different age groups, reflecting distinctions in career development stages, personal priorities, and accumulated experiences. Early-career educators place greater emphasis on intrinsic motivations (Svartefoss et al., 2024) and social influences as they shape their professional identities, factors that can substantially affect their teaching performance. Additionally, younger teachers tend to be more vulnerable to negative social dissuasion due to increased sensitivity to external criticism, which can adversely impact their instructional effectiveness. By contrast, satisfaction with choice to pursue teaching generally stabilizes with age, contributing to more consistent teaching effectiveness among veteran educators. Shrestha (2019) found that job satisfaction is typically higher among older teachers.

By contrast, Masath (2015) mentioned growing dissatisfaction in the teaching career among younger teachers. Van Droogenbroeck et al. (2014) also indicated that older teachers were less emotionally exhausted compared to their younger peers. Therefore, this study proposes the following hypothesis:

**H12.**  *Age moderates the influence of proposed predictors on teacher agency.*

**Figure 1:**
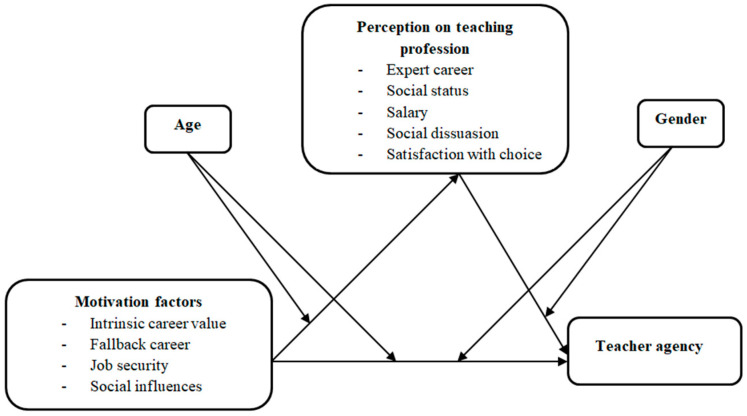
Conceptual framework.

Based on the reviewed literature, the conceptual framework for this research is depicted in Figure 1.

## 4. Materials and Methods

### 4.1. Participants and Procedure

This study targeted full-time teachers from basic schools in the Bono Region of Ghana. That is, only teachers within the basic education level, comprising kindergarten, primary, and junior high school, were included in the sample. A random selection of 20 schools was made, and data were collected via online questionnaires distributed to full-time teachers. The questionnaire link was sent to school principals, who shared it with the teachers through staff meetings and internal messaging systems. A total of 580 responses were received, but 6 were excluded due to incomplete data, leaving 574 valid responses for analysis.

The participants were assured of confidentiality, anonymity, and privacy, with their data used solely for this study. Table 1 shows that 61% of respondents were male and 39% were female. A substantial portion of the respondents (43.6%) were aged 21 to 30 years, and 49.8% were married. Additionally, 45.1% held a bachelor’s degree, and 44.8% had 1 to 5 years of work experience.

### 4.2. Measures

Teacher agency was assessed using a 24-item scale developed by Shen (2015) and adapted in several studies, including Liu et al. (2016), Bellibaş et al. (2020), and Polatcan et al. (2023). The scale includes statements like, “I am confident that I can find effective teaching methods to develop my students.” The scale, based on a 5-point Likert scale, covers four dimensions: learning effectiveness (6 items), teaching effectiveness (7 items), optimism (5 items), and constructive engagement (6 items). The inter-item reliability was strong, with a Cronbach’s alpha of 0.874.

Motivating factors were assessed using the factors influencing teaching choice (FIT-Choice) scale by Watt and Richardson (2007), which evaluates the teachers’ motivation and perceptions of the profession. The 38 items, such as “Teaching is a career suited to my abilities”, had Cronbach’s alpha values ranging from 0.67 to 0.85, indicating acceptable reliability.

Perception factors related to the teachers’ beliefs about the profession and their decision to stay were measured using 20 items, including “Do you think teachers need high levels of technical knowledge?” Rated on a seven-point Likert scale, the reliability of the perception factors was confirmed with Cronbach’s alpha values between 0.79 and 0.84.

## 5. Results

### 5.1. Preliminary Analyses

Confirmatory factor analysis (CFA) was used to evaluate the construct validity of the measurement model, employing fit indices, such as the incremental fit index (IFI), Tucker–Lewis index (TLI), comparative fit index (CFI), root mean square error of approximation (RMSEA), standardized root mean square residual (SRMR), PClose, and chi-squared (χ^2^). Model fit was considered acceptable with CFI > 0.90, SRMR < 0.08, and RMSEA < 0.06 (Hu & Bentler, 1999).

The measurement model was evaluated using a three-step approach. We initially estimated Cronbach’s alpha coefficients to assess the internal consistency of the main variables and subscales (Hair et al., 2013). Table 2 shows that the alpha coefficients for the measured variables exceeded the required threshold of 0.70 (Nunnally & Bernstein, 1994), except for job security, which had an acceptable coefficient of 0.6. In the second phase, CFA was used to assess factor loadings and average variance extracted (AVE) for the respective scales. As shown in Table 2, all subscales had acceptable factor loadings of 0.60 or higher (Hair et al., 2013). This study further showed that the AVE for the main variables exceeded 0.4, confirming convergent validity (Khan et al., 2016). Additionally, the model fit data was excellent for all three primary variables:

- Teacher agency (χ^2^/df = 4.778, *p* = 0.00, IFI = 0.977, TLI = 0.962, CFI = 0.977, RMSEA = 0.081, SRMR = 0.033, PClose = 0.016);
- Motivation factors (χ^2^/df = 3.394, *p* = 0.00, IFI = 0.958, TLI = 0.939, CFI = 0.958, RMSEA = 0.065, SRMR = 0.057, PClose = 0.024);
- Perception factors (χ^2^/df = 2.436, *p* = 0.00, IFI = 0.972, TLI = 0.961, CFI = 0.971, RMSEA = 0.050, SRMR = 0.042, PClose = 0.480).

Finally, we analyzed the data fit for the overall model. As shown in Table 2, all of the model-fit indices stated earlier were considered acceptable (χ^2^/df ratio = 2.316 (*p* < 0.001), IFI = 0.938, TLI = 0.925, CFI = 0.937, RMSEA = 0.048, SRMR = 0.048, PClose = 0.794). Therefore, we determined that the measurement model achieved the desired criteria for reliability and validity.

Table 3 shows the results of comparing the squared individual inter-construct correlations with the AVE values related to each construct. This comparison assesses discriminant validity (Fornell & Larcker, 1981). All the AVE values were greater than the square of each inter-construct correlation, meaning they met the criteria for discriminant validity. The heterotrait–monotrait (HTMT) ratio of correlations for each construct is below the threshold of 0.85 or 0.9, which indicates discriminant validity. This information can be found in Table 4.

### 5.2. Hypotheses Testing

This study employed SEM to examine the influence of the motivation and perception factors on teacher agency. Both the motivation and perception were modeled as latent constructs, each comprising multiple subscales. The motivation construct included the subscales intrinsic career value, fallback career choice, job security, and social influences. The perception construct consisted of expertise, social status, salary, social dissuasion, and satisfaction with choice. The analysis focused on the individual subscales rather than the overall effects of the higher-order latent constructs. It provides a more detailed insight into how the specific aspects of motivation and perception contribute to teacher agency, and determine the most impactful factors. Direct relationships were tested, and the findings are presented in Table 5. The results showed that intrinsic career value significantly influenced teacher agency (β = 0.449, *p* < 0.001). Fallback career (β = 0.115, *p* < 0.01), job security (β = 0.150, *p* < 0.001), and social influences (β = −0.315, *p* < 0.001) also had significant effects. Expertise in one’s work (β = 0.156, *p* < 0.001), social status (β = 0.178, *p* < 0.001), and salary (β = −0.157, *p* < 0.001) had a significant impact on teacher agency. Social dissuasion (β = 0.265, *p* < 0.001) and satisfaction with choice (β = 0.118, *p* < 0.01) also significantly impacted teacher agency. All the variables proposed in this study were confirmed to have significant effects on teacher agency in Ghana. This study analyzed the standardized regression coefficients for each determinant, with intrinsic career value having the most significant influence on teacher agency, followed by social dissuasion, social status, expert career, job security, satisfaction with choice, fallback career, salary, and social influences.

### 5.3. Mediation Analysis

**Figure 2:**
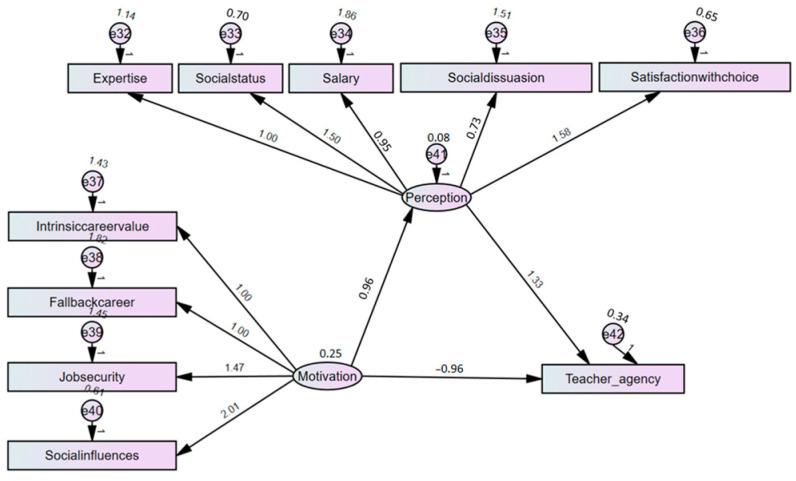
Results of the structural model for mediation analysis.

A mediation analysis using a bootstrap sample of 2000 with a 95% bias-corrected confidence interval revealed a partial mediation relationships. The findings in Table 6 show a significant indirect effect of motivation factors on teacher agency (b = 1.273, *p* = 0.001), supporting hypothesis H10. Additionally, motivation factors had a significant negative impact on perception factors in the presence of the mediator (b = −0.958, *p* = 0.001). This confirms that perception partially mediates the association between motivation and teacher agency, as seen in Table 6 and visually represented in Figure 2.

### 5.4. Moderating Influence of Gender and Age

A multi-group path analysis was conducted to explore how gender and age moderate the associations between the proposed predictors and teacher agency. Standardized path coefficients were estimated separately for male and female teachers, and z-tests were used to assess the significance of group differences. The findings revealed that the association between intrinsic career value and teacher agency was significantly stronger for female teachers (β = 0.280, *p* < 0.001) compared to male teachers (β = 0.189, *p* < 0.001), with a z-score of 2.466 (*p* < 0.05). This indicates the statistically significant moderating effect of gender, suggesting that intrinsic motivations for choosing a teaching career contribute more strongly to female teachers.

For the remaining predictors, although several showed statistically significant effects within both gender groups, such as job security (male: β = 0.065, *p* = 0.001; female: β = 0.067, *p* = 0.015), social influences, expert career, social status, social status, salary, and social dissuasion, the z-scores comparing these paths across gender were all below the critical value of ±1.96, indicating that the differences between male and female teachers were not statistically significant. For example, the path from job security to teacher agency had a z-score of 0.072, reflecting no meaningful difference between the groups. Additionally, regarding satisfaction with choice, the path was significant in one group (male: β = 0.072, *p* = 0.004) but not in the other (female: β = 0.037, *p* = 0.228). However, the z-score for this difference was −0.876, which is not statistically significant and, therefore, does not support the moderating effect of gender. These results indicate that gender moderates only the relationship between intrinsic career value and teacher agency. The path coefficients and z-scores are presented in Table 7.

Concerning age, the sample was divided into four age groups: 21–30, 31–40, 41–50, and 51–60 years. Standardized path coefficients and t-values were estimated separately for each group, and chi-squared difference tests were used to assess the statistical significance of differences across the groups. The results indicated the significant moderating effect of age on the relationship between intrinsic career value and teacher agency. Specifically, intrinsic career value had the strongest positive effect on teacher agency among the teachers aged 31–40 (β = 0.504, t = 9.034, *p* < 0.001), followed by the 41–50 (β = 0.498, t = 7.195, *p* < 0.001) and 51–60 (β = 0.451, t = 4.790, *p* < 0.001) groups. The effect was relatively weaker but still significant for the youngest group aged 21–30 (β = 0.291, t = 5.487, *p* < 0.001). No other paths demonstrated significant moderation by age. The detailed path coefficients, t-values, and chi-squared difference statistics are presented in Table 8.

## 6. Discussion

This study provides empirical findings on basic school teachers’ agency by (1) determining the association between teacher agency, motivation, and perception, (2) exploring whether gender and age moderate the influence of the proposed predictors on teacher agency, and (3) examining how perception mediates the relationship between motivation and teacher agency.

The findings suggested that intrinsic career value (β = 0.449, *p* < 0.001), fallback career (β = 0.115, *p* < 0.01), job security (β = 0.150, *p* < 0.001), and social influences (β = −0.315, *p* < 0.001) play significant roles in shaping teacher agency. The findings demonstrate that the reasons behind teachers entering the profession, either out of personal interest, practical reasons, or social influence, have a core impact on their ability to perform with agency in their teaching careers. The impact of intrinsic value of career reflects that the teachers who consider teaching a significant and personally rewarding career are likely to demonstrate autonomy and proactive initiative in teaching, aligning with Fathi et al.’s (2023) assertion that intrinsic motivation generates teacher enthusiasm and excellence. The positive impact of fallback career implies that the teachers entering the profession as an alternative may still participate actively and positively, possibly leveraging transferable skills, as argued by Agaj et al. (2023). The overarching contribution of job security also implies that perceived stability within the profession may enhance the teachers’ confidence and preparedness in making independent instructional decisions, supporting the findings by Kumar (2023). Additionally, the social influence supports the importance of family and community support in shaping the teachers’ professional agency and commitment, echoing Meyer and Turner’s (2006) emphasis on teamwork and trust in fostering effective teaching environments. These findings support the research study.

In addition, expert career (β = 0.156, *p* < 0.001), social status (β = 0.178, *p* < 0.001), salary (β = −0.157, *p* < 0.001), social dissuasion (β = 0.265, *p* < 0.001), and satisfaction with choice (β = 0.118, *p* < 0.01) positively affect teacher agency. These results underscore the significance of the teachers’ attitudes regarding the profession as a determinant of their readiness and capability to function autonomously and responsively in their professional environments. The effect of an expert career is that, if the teachers consider teaching to be a professional and intellectually demanding profession, they are most likely to demonstrate agency in the form of informed decision-making and teacher autonomy. This finding aligns with Loughran (2012), who emphasized that deep subject knowledge enhances the teachers’ confidence and quality of teaching. In the same way, the association between social status and teacher agency implies that social recognition reinforces the teachers’ professional identity and self-worth, as proposed by Hagenauer et al. (2023). The function of salary conforms to the perception that adequate financial compensation contributes to teacher motivation and long-term professional involvement, consistent with the findings of Ololube (2006). Interestingly, social dissuasion, often regarded as a type of deterrence, was found to have been positively related to teacher agency, implying that social skepticism or discouragement actually pushes some teachers to think critically about their professional position and use their agency more consciously, as implied by Eren and Çetin (2019). Finally, satisfaction with opting to become a teacher also contributed positively to teacher agency, consistent with Nida and Ningsih’s (2023) conclusion that personal satisfaction with one’s job augments participation as well as teaching quality. The results corroborate the research findings.

The moderation analysis revealed that intrinsic career value was a significant predictor of teacher agency for both the male (β = 0.189, *p* < 0.001) and the female teachers (β = 0.280, *p* < 0.001), with a more substantial impact observed among the females. This finding corroborates Nagy et al. (2008), who reported that women place greater value on intrinsic motivations for choosing teaching careers. It was the only predictor for which gender showed a statistically significant moderating effect, indicating that intrinsic motivations contribute more strongly among female teachers than among their male counterparts.

Although job security, social influences, social status, salary, social dissuasion, and satisfaction with choice were significant predictors within one or both gender groups, none of these paths showed a statistically significant difference between males and females (i.e., non-significant z-scores). As such, while some gender patterns align with the prior literature (Ellemers, 2018; Jugovic et al., 2022; Hyllegard et al., 2017; Han et al., 2020), these results do not support gender as a moderator for these variables in the current study.

Our findings also suggest that age has a significant moderating effect on the relationship between intrinsic career value and teacher agency, with the most significant effects observed for older and mid-career teachers. Specifically, intrinsic career value had the strongest positive effect on teacher agency among the teachers aged 31–40 (β = 0.504, t = 9.034, *p* < 0.001), followed by the 41–50 (β = 0.498, t = 7.195, *p* < 0.001) and 51–60 (β = 0.451, t = 4.790, *p* < 0.001) groups. The effect was relatively weaker but still significant for the youngest group aged 21–30 (β = 0.291, t = 5.487, *p* < 0.001). This indicates that these age groups of teachers place greater value on intrinsic reasons for teaching. This result aligns with Wyatt-Smith et al. (2017), who established that older teachers have higher scores on intrinsic career value than younger teachers.

Finally, perception factors mediate the relationship between motivation and teacher agency. Akinci et al. (2018) reported that perceptions of academic education influence satisfaction with education sustainability, while Ahmadi Deh Qutbuddini et al. (2022) confirmed that perception plays a critical role in mediating ICT application in education. These findings affirm this study’s conclusion that perception partially mediates the interaction between motivation and teacher agency.

### Implications

This study fills a gap in understanding these relationships in Ghana by applying SDT, providing new insights into gender and age differences and the mediating role of perception. It also supports the previous research and demonstrates the effectiveness of SEM in examining complex relationships between variables.

In practice, the findings can inform the Ghanaian Ministry of Education, teacher educators, and policymakers, emphasizing the importance of improving teacher remuneration, empowering teachers in decision-making, and enhancing the profession’s prestige. By addressing perception factors, policymakers can create a supportive environment that fosters teacher agency and motivation, ultimately improving teaching and learning outcomes.

While this study offers meaningful insights into teacher agency within the Ghanaian context, several limitations should be acknowledged to guide future research. First, the data were obtained exclusively from teachers in Ghana, which may constrain the broader applicability of the findings across different educational systems and cultural settings. Second, the use of a cross-sectional research design limits the ability to examine how teacher agency, motivation, and perceptions of the teaching profession evolve. To address this, future studies should adopt longitudinal approaches, particularly within both African and international contexts, to capture the dynamic nature of these constructs.

Moreover, further research should broaden the scope by including teachers from private basic schools and extending the data collection across multiple regions to enhance representativeness. It is also crucial to explore how school-level policies and institutional environments influence teachers’ long-term commitment to the profession. In particular, factors such as working conditions and job satisfaction warrant closer examination, as they play a critical role in shaping teachers’ professional engagement and retention. This study provides a foundational basis for subsequent inquiries into educational reform and teacher development within Ghana’s basic education landscape.

## 7. Conclusions

The current research examined teacher agency and its effects on the motivation and perceptions of the teaching profession. It was found that intrinsic career value, fallback career, job security, and social influences positively affected teacher agency. Variables such as expert career, social status, salary, social dissuasion, and satisfaction with choice also have a highly significant effect on teacher agency. The moderator analysis revealed that intrinsic career value was a significant predictor of teacher agency for both male and female teachers. The results also indicated a significant moderating effect of age on the relationship between intrinsic career value and teacher agency. Perceptions partially mediated the relationship between motivation and teacher agency.

## Figures and Tables

**Table 1:** Participants information (*N* = 574).

Variables		Frequency (*n*)	Percent (%)
Gender			
	Male	350	61.0
	Female	224	39.0
Age			
	21–30	250	43.6
	31–40	179	31.2
	41–50	93	16.2
	51–60	52	9.1
Marital status			
	Married	286	49.8
	Divorced	25	4.4
	Single	239	41.6
	Separated	24	4.2
Academic/Professional Qualifications			
	Diploma	256	44.6
	Bachelor’s degree	259	45.1
	Master’s degree	59	10.3
Number of years in service			
	1–5	257	44.8
	6–10	160	27.9
	11–15	83	14.5
	Above 16	74	12.9

**Table 2:** Reliability and convergent validity of the measurement model.

Construct and Measurement Items	β	t-Value (Significance)	CR	AVE	α
Teacher agency (TA)			0.875	0.538	0.874
TE1	0.698	15.039 ***			
TE2	0.787	16.724 ***			
TE3	0.727	15.609 ***			
TE4	0.754	16.115 ***			
TE5	0.744	15.927 ***			
TE6	0.686	Fixed			
**Motivation factors**					
Intrinsic Career Value (I.C.)			0.853	0.660	0.851
IF7	0.808	Fixed			
IF1	0.766	19.462 ***			
IF11	0.861	22.102 ***			
Fallback career (F.C.)			0.751	0.501	0.752
IF27	0.748	Fixed			
IF10	0.683	13.787 ***			
IF35	0.692	13.923 ***			
Job security (J.S.)			0.672	0.508	0.666
IF23	0.772	Fixed			
IF30	0.648	12.459 ***			
Social influences (SI)			0.725	0.468	0.724
IF21	0.682	Fixed			
IF3	0.659	13.383 ***			
IF32	0.709	14.197 ***			
**Perception factors**					
Expertise (E.C.)			0.842	0.640	0.841
BT9	0.791	19.603 ***			
BT14	0.796	19.709 ***			
BT13	0.814	Fixed			
Social Status (S.S.)			0.808	0.513	0.807
BT11	0.701	14.907 ***			
BT7	0.712	15.122 ***			
BT12	0.749	15.800 ***			
BT8	0.701	Fixed			
Salary (S)			0.832	0.713	0.832
BT3	0.820	19.307 ***			
BT1	0.868	Fixed			
Social Dissuasion (SD)			0.798	0.570	0.790
TD2	0.639	14.363 ***			
TD6	0.802	16.929 ***			
TD4	0.812	Fixed			
Satisfaction with Choice (SWC)			0.794	0.658	0.792
TD5	0.778	18.339 ***			
TD3	0.843	Fixed			

Note: *n* = 574; *** *p* ≤ 0.001; if CR > 0.70; if AVE > 0.40; if factor loading > 0.50.

**Table 3:** Descriptive statistics, correlations, and discriminant validity.

Variables	M	SD	1	2	3	4	5	6	7	8	9	10
1. IC	4.70	1.32	0.813									
2. FC	3.99	1.41	−0.269 ***	0.708								
3. JS	4.83	1.41	0.008	0.568 ***	0.713							
4. SI	4.25	1.27	0.481 ***	0.406 ***	0.570 ***	0.684						
5. EC	5.46	1.21	0.492 ***	−0.168 **	0.240 ***	0.176 **	0.800					
6. SS	4.60	1.19	0.404 ***	0.175 **	0.439 ***	0.644 ***	0.426 ***	0.716				
7. S	3.86	1.47	0.050	0.304 ***	0.266 ***	0.505 ***	−0.250 ***	0.578 ***	0.844			
8. SD	4.91	1.30	−0.065	0.358 ***	0.430 ***	0.115 *	0.262 ***	0.205 ***	0.093 ^†^	0.755		
9. SWC	4.88	1.20	0.617 ***	0.007	0.486 ***	0.627 ***	0.489 ***	0.620 ***	0.301 ***	0.247 ***	0.811	
10. TA	3.91	0.72	0.486 ***	0.007	0.258 ***	0.086	0.511 ***	0.302 ***	−0.137 **	0.365 ***	0.429 ***	0.734

Note: IC = Intrinsic career value; FC = Fallback career; JS = Job security; SI = Social influences; EC = Expertise; SS = Social status; S = Salary; SD = Social dissuasion; SWC = Satisfaction with choice; TA = Teacher agency; ^†^ *p* < 0.100, * *p* < 0.05, ** *p* < 0.01, *** *p* < 0.001.

**Table 4:** HTMT Analysis for Discriminant Validity.

Construct	1	2	3	4	5	6	7	8	9	10
1. Intrinsic career value	_									
2. Fallback career	0.266	_								
3. Job security	0.045	0.570	_							
4. Social influences	0.478	0.396	0.562	_						
5. Expert	0.495	0.166	0.250	0.181	_					
6. Social status	0.402	0.169	0.418	0.645	0.426	_				
7. Salary	0.044	0.296	0.228	0.513	0.240	0.585	_			
8. Social dissuasion	0.061	0.392	0.457	0.130	0.280	0.212	0.097	_		
9. Satisfaction with choice	0.628	0.002	0.501	0.631	0.495	0.615	0.292	0.251	_	
10. Teacher agency	0.492	0.015	0.308	0.087	0.512	0.305	0.129	0.380	0.430	_

Note: The HTMT ratio of correlations for each construct is below the threshold of 0.85 or 0.9, confirming discriminant validity.

**Table 5:** Direct path analysis.

Hypothesis	Path Description	β	t-Value (Significance)	Results
	Motivation factors			
H1	Intrinsic career value → Teacher agency	0.449	9.866 ***	Supported
H2	Fallback career → Teacher agency	0.115	2.785 **	Supported
H3	Job security → Teacher agency	0.150	4.792 ***	Supported
H4	Social influences → Teacher agency	−0.315	−6.635 ***	Supported
	Perception factors			
H5	Expert career → Teacher agency	0.156	3.938 ***	Supported
H6	Social status → Teacher agency	0.178	4.349 ***	Supported
H7	Salary → Teacher agency	−0.157	−4.193 ***	Supported
H8	Social dissuasion → Teacher agency	0.265	6.072 ***	Supported
H9	Satisfaction with choice → Teacher agency	0.118	2.742 **	Supported

Note: ** *p* < 0.01, *** *p* < 0.001 (N = 574).

**Table 6:** Results of mediation analysis.

Effect	Path	β	Confidence Interval		Conclusion
			Lower Bound	Upper Bound	
Total	Motivation→ teacher agency	0.314 *	0.087	0.499	
Direct	Motivation→ teacher agency	−0.958 **	−3.888	−0.497	
Indirect	Motivation → perception → teacher agency	1.273 ***	0.066	0.271	Partial Mediation

Note: * *p* < 0.05, ** *p* < 0.01, *** *p* ≤ 0.001.

**Table 7:** Results of moderation analysis (Gender).

	Male		Female			
Path Description	β	*p*	β	*p*	z-Score	Results
Intrinsic career value → Teacher agency	0.189	0.000	0.280	0.000	2.466 **	Supported
Fallback career → Teacher agency	0.034	0.088	0.050	0.078	0.472	Not supported
Job security → Teacher agency	0.065	0.001	0.067	0.015	0.072	Not supported
Social influences → Teacher agency	−0.128	0.000	−0.158	0.000	−0.792	Not supported
Expert career → Teacher agency	0.060	0.017	0.092	0.002	0.832	Not supported
Social status → Teacher agency	0.066	0.005	0.093	0.005	0.644	Not supported
Salary → Teacher agency	−0.070	0.000	−0.065	0.013	0.145	Not supported
Social dissuasion → Teacher agency	0.139	0.000	0.114	0.000	−0.689	Not supported
Satisfaction with choice → Teacher agency	0.072	0.004	0.037	0.228	−0.876	Not supported

Note: ** *p*-value < 0.05.

**Table 8:** Results of moderation analysis (Age).

	Age 21–30	Age 31–40	Age 41–50	Age 51–60		
Path Description	β Value (t-Value)	β Value (t-Value)	β Value (t-Value)	β Value (t-Value)	χ^2^ Difference	Result
Intrinsic career value → Teacher agency	0.291 (5.487 ***)	0.504 (9.034 ***)	0.498 (7.195 ***)	0.451 (4.790 ***)	10.321 *	Supported
Fallback career → Teacher agency	0.020 (0.382)	0.190 (3.400 ***)	0.079 (1.146)	0.108 (1.150)	3.819	Not supported
Job security → Teacher agency	0.054 (1.017)	0.128 (2.291 *)	0.219 (3.160 **)	0.232 (2.463 *)	3.414	Not supported
Social influences → Teacher agency	−0.247 (−4.661 **)	−0.253 (−4.543 ***)	−0.286 (−4.132 ***)	−0.352 (−3.732 ***)	1.154	Not supported
Expert career → Teacher agency	0.105 (1.988 *)	−0.043 (−0.762)	0.240 (3.459 ***)	0.216 (2.294 *)	5.682	Not supported
Social status → Teacher agency	0.175 (3.303 ***)	0.141 (2.526 *)	0.102 (1.476)	0.079 (0.836)	0.199	Not supported
Salary → Teacher agency	−0.135 (−2.539 *)	−0.207 (−3.711 ***)	−0.152 (−2.194 *)	−0.069 (−0.733)	1.402	Not supported
Social dissuasion → Teacher agency	0.246 (4.637 ***)	0.105 (1.877)	0.241 (3.480 ***)	0.300 (3.184 ***)	3.324	Not supported
Satisfaction with choice → Teacher agency	0.182 (3.424 ***)	0.023 (0.406)	0.153 (2.203 *)	0.020 (0.211)	1.979	Not supported

Note: * *p* < 0.05, ** *p* < 0.01, *** *p* < 0.001.

## Data Availability

The original contributions presented in this study are included in the article. Further inquiries can be directed to the corresponding author.
